# Repetitive temporal interference stimulation improves jump performance but not the postural stability in young healthy males: a randomized controlled trial

**DOI:** 10.1186/s12984-024-01336-7

**Published:** 2024-03-20

**Authors:** Suwang Zheng, Tianli Fu, Jinlong Yan, Chunyue Zhu, Lu Li, Zhenyu Qian, Jiaojiao Lü, Yu Liu

**Affiliations:** 1 Key Laboratory of Exercise and Health Sciences of Ministry of Education, Shanghai University of Sport, Shanghai, 200438 China; 2 School of Exercise and Health, Shanghai University of Sport, Shanghai, 200438 China

**Keywords:** Temporal interference stimulation, Non-invasive brain stimulation, Lower limbs motor function, Vertical jump, Postural stability

## Abstract

**Background:**

Temporal interference (TI) stimulation, an innovative non-invasive brain stimulation technique, has the potential to activate neurons in deep brain regions. The objective of this study was to evaluate the effects of repetitive TI stimulation targeting the lower limb motor control area (i.e., the M1 leg area) on lower limb motor function in healthy individuals, which could provide evidence for further translational application of non-invasive deep brain stimulation.

**Methods:**

In this randomized, double-blinded, parallel-controlled trial, 46 healthy male adults were randomly divided into the TI or sham group. The TI group received 2 mA (peak-to-peak) TI stimulation targeting the M1 leg area with a 20 Hz frequency difference (2 kHz and 2.02 kHz). Stimulation parameters of the sham group were consistent with those of the TI group but the current input lasted only 1 min (30 s ramp-up and ramp-down). Both groups received stimulation twice daily for five consecutive days. The vertical jump test (countermovement jump [CMJ], squat jump [SJ], and continuous jump [CJ]) and Y-balance test were performed before and after the total intervention session. Two-way repeated measures ANOVA (group × time) was performed to evaluate the effects of TI stimulation on lower limb motor function.

**Results:**

Forty participants completed all scheduled study visits. Two-way repeated measures ANOVA showed significant group × time interaction effects for CMJ height (*F* = 8.858, *p* = 0.005) and SJ height (*F* = 6.523, *p* = 0.015). The interaction effect of the average CJ height of the first 15 s was marginally significant (*F* = 3.550, *p* = 0.067). However, there was no significant interaction effect on the Y balance (*p* > 0.05). Further within-group comparisons showed a significant post-intervention increase in the height of the CMJ (*p* = 0.004), SJ (*p* = 0.010) and the average CJ height of the first 15 s (*p* = 0.004) in the TI group.

**Conclusion:**

Repetitive TI stimulation targeting the lower limb motor control area effectively increased vertical jump height in healthy adult males but had no significant effect on dynamic postural stability.

## Background

Physical competence plays a pivotal role in patient rehabilitation, athletes' competitive prowess, adolescent development, and the quality of life among the elderly. Recently, non-invasive brain stimulation (NIBS), such as transcranial direct current stimulation (tDCS) and transcranial alternating current stimulation (tACS), have been widely studied in sports science and rehabilitation medicine to improve human motor ability [1–3]. However, many motor-related brain areas, such as basal ganglia, are located in the deep cortex or nuclei [4, 5], which necessitates greater requirements in terms of depth and focus of stimulation.

Temporal interference (TI) stimulation is an innovative neuromodulation technique, which delivers alternating currents of two distinct frequencies (> 1 kHz) to the brain from different positions on the scalp. These two high-frequency currents overlap within the brain tissue, generating an low-frequency envelope wave at the frequency difference [6]. Neurons selectively respond to the low-frequency envelope wave while disregarding the high-frequency carrier waves given its nonlinear characteristics and low-pass filtering properties [6]. Theoretically, TI has the potential to activate neurons in deep brain regions without engaging cortical neurons [7], which offers a promising avenue that overcomes the limitations of conventional transcranial electrical stimulation, including superficial stimulation and poor focus. However, it is important to note that the current status of this technology is exploratory; accordingly, further research and validation are warranted to establish its theoretical credibility, technical feasibility, and parameter standardization.

Since 2017, several global research teams have validated and applied TI technology from various perspectives, including electric field simulation [8–10], animal experiments [11–13], cadaver studies [11, 14], and human trials [15–17]. Although computational simulations and animal studies have confirmed the effectiveness of this technology, there is a paucity of human trials specifically related to motor abilities. Most studies have primarily focused on stimulating superficial brain regions to assess the safety of TI-mediated modulation of the human brain. Ma et al. reported the positive impact of 20-Hz TI stimulation targeting the primary motor cortex (M1) on motor learning in healthy young participants [15]. This finding demonstrated that TI stimulation facilitates human motor function and motor cortex excitability. A neuroimaging study indicated a significant enhancement in functional connectivity strength between M1 and secondary motor areas (premotor and supplementary motor areas) following TI stimulation [18], which provide additional evidence regarding the potential of TI stimulation to modulate human motor performance.

However, these studies exclusively validated TI stimulation protocols targeting the superficial upper limb motor control areas [15, 17, 19]. Compared with these superficial cortical regions, the lower limb motor control area, which is located in the longitudinal fissure, represents a relatively deeper target region associated with motor abilities. Therefore, this study aimed to utilize repetitive TI stimulation to target the lower limb motor control area (i.e., the M1 leg area) and to investigate the modulation effects of this technique on lower limb motor function in healthy individuals, which could provide evidence for further translational application of non-invasive deep brain stimulation. We hypothesized that (1) repetitive TI stimulation would improve vertical jump height and the corresponding biomechanical indexes compared with sham stimulation and (2) repetitive TI stimulation would improve postural stability of young healthy males.

## Methods

### Experiment design

**Fig. 1:**
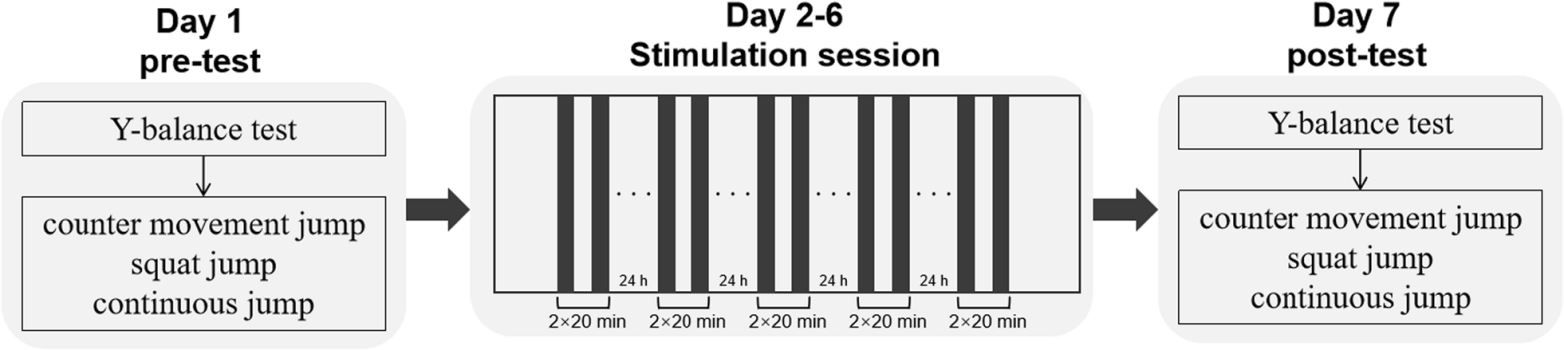
Study design

In this randomized, double-blinded, parallel-controlled trial, participants were randomly assigned to either the TI group (TI stimulation) or the sham group (sham stimulation). At the first visit, we collected the participants’ anthropometric data (age, height, weight, and leg length) and baseline assessments of lower limb motor abilities (Y-balance, countermovement jump [CMJ], squat jump [SJ], and continuous jump [CJ]). Subsequent visits involved a continuous 5-day intervention followed by completion of a questionnaire on blinding efficacy and side effects. On the second day following the completion of all stimulation sessions, participants revisited the laboratory for re-evaluation of lower limb motor abilities (study design, see Fig. 1). This study was conducted at the Biomechanics Laboratory of Shanghai University of Sport from September 2021 to November 2022. The study successfully achieved the anticipated sample size, and all scheduled interventions and assessments were conducted according to the predetermined plan.

### Participants

**Fig. 2:**
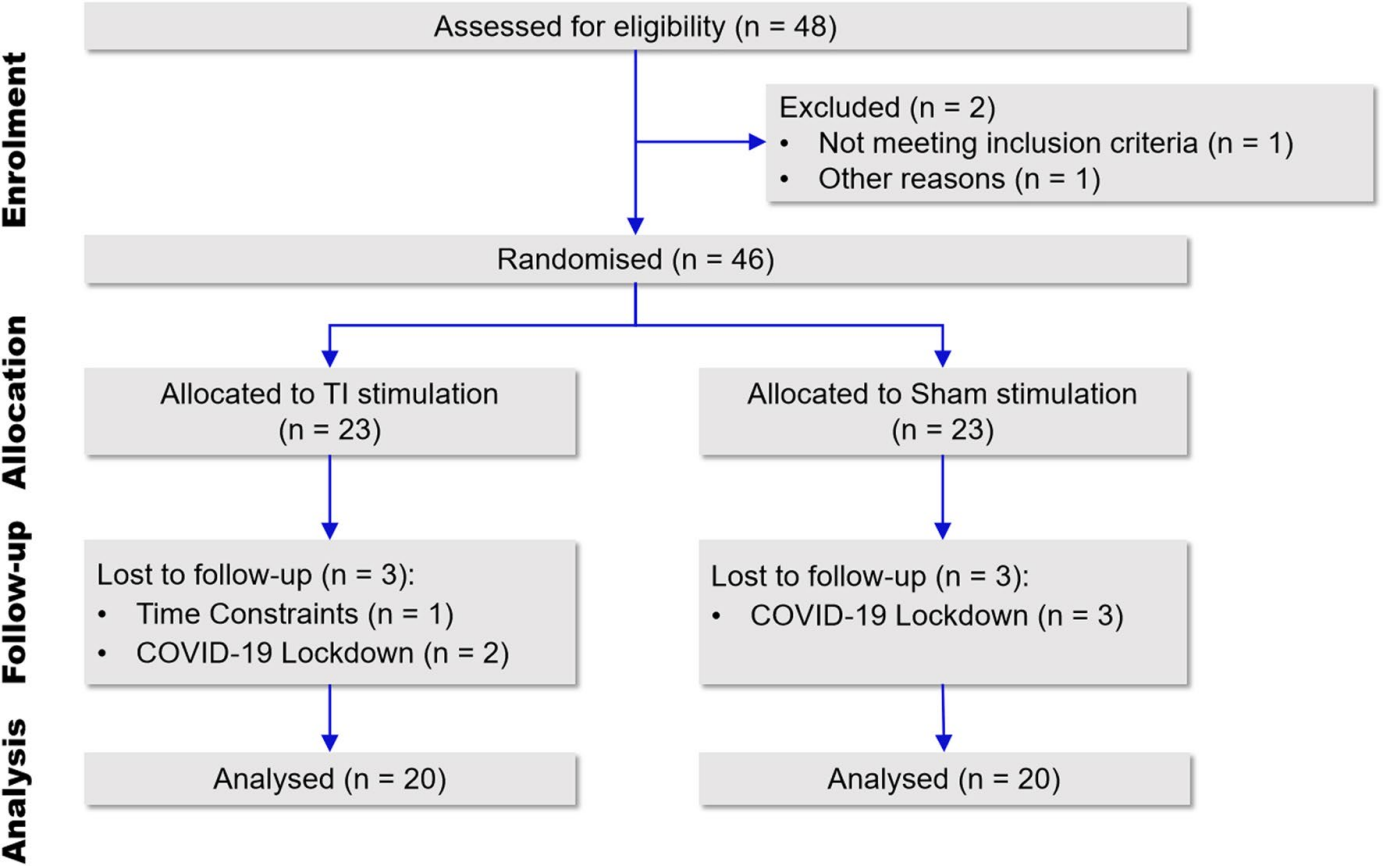
Participant flow diagram

Forty-six healthy adult males were recruited; among them, forty participants completed this study (participant flow diagram, see Fig. 2). The inclusion criteria were as follows: (1) good physical condition, and ability to complete a 60-s continuous vertical jump test; (2) right-handedness with the right leg as the dominant leg; and (3) no lower limb injuries within the past 3 months. The exclusion criteria were as follows: (1) personal or family history of neurological diseases; (2) participation in another experiment involving non-invasive brain stimulation or strength training; (3) history of lower limb injuries; (4) presence of metallic implants in the head; and (5) receipt of invasive treatment within the past 6 weeks.

Before enrollment, all participants were required to understand the study’s purpose, experimental procedures, and precautions. All participants provided written informed consent during the first visit. Ethical approval was granted by the Institutional Review Board of the Shanghai University of Sport (102772022RT051).

### Procedures

#### Stimulation paradigm

The custom-made Temporal Interference Stimulation System utilized in this study was developed based on a study conducted by Grossman et al. [6]. It comprised multiple components, including MATLAB programs, converters, and isolators [6]. The MATLAB program tailored for this study generated digital signals for TI stimulation. Subsequently, these signals were output through a converter (USB-6361, National Instruments Inc., America) and delivered as electrical currents via an A395 linear stimulus isolator (A395, World Precision Instruments Inc., America) [17].

**Fig. 3:**
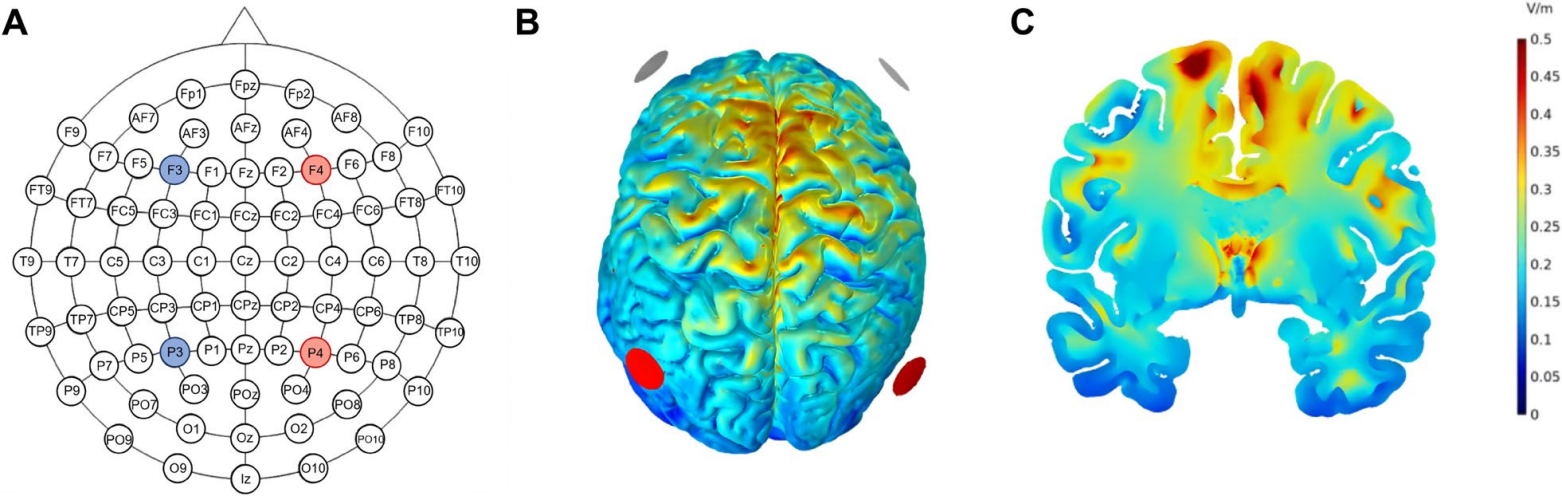
Electrode placement and the distribution of the envelope electric field. **A** The red and blue circles represent the stimulation points for two alternating currents; red represents one channel of alternating current, while blue represents the other channel. **B**, **C** Illustrates the envelope electric field distribution on the cortical surface and coronal plane, respectively. The shades of red and blue denote different electric field intensities, with a stronger intensity being indicated by a shift toward the red color

The TI stimulation electrodes were placed at F3, P3, F4, and P4 (based on the International 10–10 Electroencephalography System [20], Fig. 3A). Specifically, F3 and P3 were designated as one alternating current, whereas F4 and P4 were allocated as another, with frequencies set at 2 kHz and 2.02 kHz (resulting in a frequency difference of 20 Hz). The target region for this stimulation paradigm was the lower limb motor control area of the primary motor cortex (the M1 leg area). The electric field simulation diagrams are shown in Fig. 3B and C. The peak-to-peak amplitude of the current was set at 2 mA. Stimulation was continuously administered twice daily for 5 consecutive days, with a 20-min interval between sessions. Each session of stimulation lasted for a total of 20 min. For the Sham group, the electrode placement, current intensity, and frequencies were identical to those of the TI group. However, the current of sham stimulation was applied only at the beginning and end of the stimulation, with a 30-s ramp-up and ramp-down, and no current input during the intervening 19 min [21].

Participants remained seated, refrained from using mobile devices, and minimized their head movements during the stimulation sessions. The experimenters closely monitored the participants and immediately interrupted the stimulation if any unusual sensations were reported. The study strictly adhered to double-blinded experimental requirements, with only one experimenter being aware of the stimulation type. The participants and assessors remained blinded to the intervention.

#### Vertical jump test

Vertical jump performance data were collected using a three-dimensional force platform (9287C, Kistler, Switzerland) at a sampling frequency of 1000 Hz. Participants were assessed for three types of vertical jumps: countermovement jump (CMJ), squat jump (SJ), and continuous jump (CJ) with a fixed order. For CMJ, the participants were instructed to rapidly move downward upon hearing a command, followed by a maximal vertical jump. After landing, the participants were required to flex their knees to absorb the impact and return to their starting position. The SJ required participants to reposition themselves in a squat and then perform a maximal jump after holding the position statically for at least 3 s. Both jumps were completed three times, and the height, ground reaction force, and impulse indices were calculated. The CJ involved participants performing CMJ for 1 min. The participants were instructed to keep their hands on their hips to eliminate the influence of upper-limb movements on jump performance. The average heights of the initial 15 s (*H*_*first15s*_) and last 15 s (*H*_*last15s*_) during CJ were calculated separately. These two measurements were used to calculate the fatigue index.

MATLAB was employed for the preprocessing and analysis of the kinetic signals. The ground reaction force was filtered at a cut-off frequency of 50 Hz. Key metrics, including jump height, impulse, and fatigue index, were calculated using the following formulae:

Jump Height: Jump height was calculated using the formula $H=\frac{1}{8} g{T}^{2}$ proposed by Yamashita et al. [22]. Based on flight time, this approach demonstrated low internal heterogeneity among participants, providing a reliable measure for assessing individual changes before and after the intervention.

Ground Reaction Force: Maximum ground reaction force during the concentric phase of the vertical jump.

Impulse: Calculated using the formula $I={\int }_{0}{\prime}F(t)dt$ where *F(t)* represents the resultant external force, and *t* is the ground contact time.

Fatigue Index (FI): Calculated as $FI=\frac{{H}_{first15s}-{H}_{last15s}}{{H}_{first15s}}\times 100\%$, where *H*_*first15s*_ is the average height of the initial 15 s and *H*_*last15s*_ is the average height of the last 15 s.

#### Y balance test

The Y-balance test was used to assess the dynamic postural stability of the dominant leg (all participants were right-legged) [23, 24]. The participants stood barefoot on their dominant leg (right leg) with their toes positioned at a marked line on a fixed platform. While maintaining a stable single-leg stance, the participants pushed a movable platform sequentially in the anterior, posteromedial, and posterolateral directions. During the test, participants were instructed to keep their hands on their hips facing forward with the non-supporting foot elevated. If the non-supporting foot touched the ground midway, the trial was considered unsuccessful and was repeated. Three successful attempts were made in each direction, and the distance (cm) was recorded. The Push Distance (% of leg length) was calculated using the following formula to standardize the data: *Push Distance (%)* = *Distance/Leg Length* × *100%,* where *Leg Length* represents the distance from the greater trochanter to the lateral malleolus (cm).

### Blinding and randomization

The random allocation sequence was generated using the SPSS (version 26.0; IBM, Armonk, NY, USA) random number generator, assigning participants to the TI group or sham group with a 1:1 allocation ratio. All data were stored on a password-protected computer; additionally, the stimulation procedure was named words unrelated to the stimulation. Recruitment personnel, data collectors, statistical analysts, and participants were blinded to the group allocation, with a designated researcher being responsible for intervention based on the group assignments.

Participants were not informed of any differences between TI and sham stimulation. Sham stimulation involve a 30-s ramp-up and ramp-down, which created an itching sensation similar to the real stimulation for the participants. To ensure successful blinding, participants were asked to guess whether the stimulation was real or sham at the end of the stimulation. Information regarding side effects, including tingling, itching, burning, skin redness, drowsiness, inattention, and mood swings, was collected using a side-effect questionnaire [25]. Participants rated the severity of each side effect as none, mild, moderate, or severe.

### Statistical analyses

The primary statistical model was a two-way repeated measures ANOVA based on the research design. The desired statistical power was set at 0.80, a significance level of 0.05, and an effect size of 0.40, resulting in a minimum sample size of 16 per group. Considering potential dropout rates, 46 participants were included in the study.

The normality of distribution was assessed using the Shapiro–Wilk normality test. Normally distributed data are presented as mean ± standard deviations (SD). Independent sample t-tests were used for between-group comparisons of anthropometric data. When the data met the assumptions of normality and homogeneity of variances, two-way repeated measures ANOVA was used to investigate the effects of group (TI vs. sham) and time (pre-test vs. post-test) on vertical jump performance and dynamic postural stability. Paired t-tests were used to assess within-group differences before and after stimulation. The significance level (α) was set at 0.05, and the effect size was represented by *η*^*2*^. Statistical analyses were performed using SPSS (version 26.0; IBM, Armonk, NY, USA).

## Results

### Baseline analyses

Forty healthy adult male participants completed the study successfully. There were no significant between-group differences in anthropometric data (Table 1). For baseline performance (pre-test), there were no significant between-group differences in lower limb vertical jump performance and dynamic postural stability, except for the SJ’s ground reaction force and the Y-balance test’s anterior distance (Table 2).

**Table 1:** Anthropometric data of participants

Variables	TI group (*n* = 20)	Sham group (*n* = 20)	*t*	*p*
Age (years)	21.750 ± 1.803	21.800 ± 2.042	− 0.082	0.629
Height (cm)	176.600 ± 5.051	178.800 ± 7.438	− 1.094	0.333
Weight (kg)	77.100 ± 15.124	79.350 ± 13.850	− 0.491	0.713
Leg length (cm)	85.983 ± 7.202	86.756 ± 5.261	− 0.426	0.673

### Countermovement jump

**Fig. 4:**
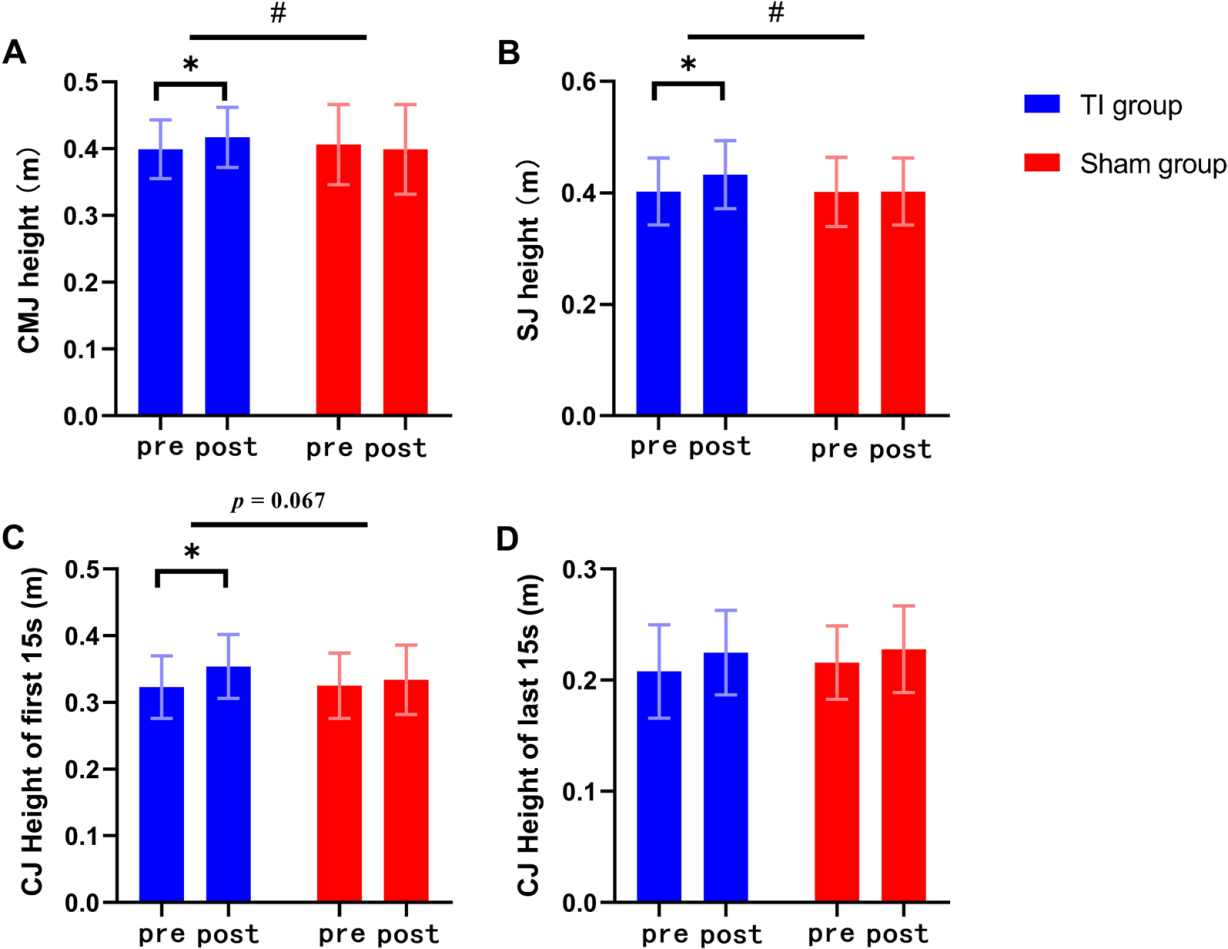
The effect of repetitive TI stimulation on vertical jump performance. Significant interaction effects of CMJ height (**A**) and SJ height (**B**) were observed. The interaction effect of CJ height of the first 15 s (**C**) was marginally significant, while no significant ineraction effect was observed in the average CJ height of the last 15 s (**D**). Within-group comparisons showed a significant post-intervention increase in the CMJ height, SJ height, and the average CJ height of the first 15s in the TI group. CMJ: countermovement jump; SJ: squat jump; CJ: continuous jump. *#* indicates a significant interaction effect (*p* < 0.05) and * indicates significant within-group differences (*p* < 0.05)

The two-way repeated measures ANOVA revealed a significant interaction effect between group and time for CMJ height (*F* = 8.858, *p* = 0.005, *η*^*2*^ = 0.189, Table 2). Within-group comparisons indicated that the TI group revealed a significant improvement in CMJ height (*t* = − 3.241, *p* = 0.004, Fig. 4A) and impulse (*t* = − 2.933, *p* = 0.009), with increases of 4.53% and 8.49%, respectively. Contrastingly, the sham group showed no significant differences before and after stimulation (*p* > 0.05).

**Table 2:** The effect of repetitive TI stimulation on vertical jump performance and dynamic postural stability

Variables	TI group		Sham group		Time × Group		
	Pre	Post	Pre	Post	*F*	*p*	*η*^2^
CMJ							
Height (m)	0.399 ± 0.044	0.417 ± 0.045^*^	0.406 ± 0.060	0.399 ± 0.067	8.858	0.005^#^	0.189
GRF (N)	1024.359 ± 385.043	1102.199 ± 466.786	1318.919 ± 507.446	1333.441 ± 437.693	0.286	0.596	0.008
Impulse (N*s)	339.072 ± 75.314	366.816 ± 89.434^*^	379.964 ± 111.208	387.405 ± 110.017	1.611	0.212	0.041
SJ							
Height (m)	0.403 ± 0.060	0.433 ± 0.061^*^	0.402 ± 0.062	0.403 ± 0.060	6.523	0.015^#^	0.147
GRF (N)^$^	1173.085 ± 270.320	1134.537 ± 275.082	1196.925 ± 464.975	1225.783 ± 405.963	1.555	0.220	0.040
Impulse (N*s)	311.185 ± 71.065	317.418 ± 83.058	305.465 ± 87.473	314.525 ± 96.865	0.051	0.823	0.001
CJ							
Height of first 15 s (m)	0.323 ± 0.047	0.354 ± 0.048^*^	0.325 ± 0.049	0.334 ± 0.052	3.550	0.067	0.085
Height of last 15 s (m)	0.208 ± 0.042	0.225 ± 0.038	0.216 ± 0.033	0.228 ± 0.039	0.149	0.702	0.004
Fatigue index (%)	35.082 ± 11.471	35.838 ± 11.264	32.433 ± 12.480	30.582 ± 15.072	0.289	0.597	0.015
Y-balance (%)							
Anterior^$^	72.500 ± 8.835	72.650 ± 9.691	66.650 ± 9.304	67.100 ± 7.166	0.317	0.577	0.009
Postero-medial	113.850 ± 13.511	116.200 ± 14.916^*^	106.550 ± 12.224	109.700 ± 9.953	0.153	0.698	0.004
Postero-lateral	122.250 ± 14.553	122.350 ± 12.787	115.650 ± 11.563	117.700 ± 10.785	1.335	0.255	0.034

CMJ: countermovement jump; SJ: squat jump; CJ: continuous jump; GRF: ground reaction force

^*#*^Indicates a significant interaction effect (*p* < 0.05), * indicates significant within-group differences (*p* < 0.05), ^$^ indicates analysis of covariance (ANCOVA), and the baseline data were incorporated as covariates

### Squat jump

For SJ height, there was a significant interaction effect between group and time (*F* = 6.523, *p* = 0.015, *η*^*2*^ = 0.147, Table 2). Subsequent within-group comparisons revealed a significant 8.01% increase in the SJ height in the TI group (*t* = − 2.854, *p* = 0.010, Fig. 4B). Contrastingly, the sham group showed no significant differences before and after stimulation (*p* > 0.05).

### Continuous jump

For the average CJ height of the first 15 s, there was a marginally significant interaction effect between group and time (*F* = 3.550, *p* = 0.067, *η*^*2*^ = 0.085, Table 2). Within-group analysis indicated a significant 10.34% post-intervention increase in the average CJ height of the first 15 s in the TI group (*t* = − 3.308, *p* = 0.004, Fig. 4C). Contrastingly, the sham group showed no significant changes (*p* > 0.05). No significant within-group differences were observed in the average CJ height of the last 15 s (*p* > 0.05, Fig. 4D).

### Dynamic postural stability

Two-way repeated measures ANOVA indicated no significant interaction effects for the Y-balance test’s anterior, posteromedial, or posterolateral reach distances (*p* > 0.05, Table 2). Within-group analysis revealed a significant 1.98% increase in posterior-medial reach distance for the TI group after stimulation (*t* = − 3.030, *p* = 0.007).

### Blinding efficacy and side effects

The TI stimulation protocol demonstrated effective blinding, with an overall correct classification rate of 52.5% for predicting the stimulation type. No severe side effects were reported in the side effect questionnaire. Only 20% of the participants reported mild-to-moderate tingling and itching, 5% reported moderate burning sensation, 20% reported moderate drowsiness, and 35% reported mild inattention during stimulation.

## Discussion

This randomized, double-blind study demonstrated that repetitive TI stimulation over lower limb motor control area (the M1 leg area) significantly improved the vertical jump performance of healthy adult males; however, we observed no effect on dynamic postural stability. Consistent with previous findings [17, 19], no serious side effects were observed during TI stimulation, indicating its safety and effectiveness.

Several studies have applied 20-Hz externally induced oscillations over M1 to influence motor performance and motor learning [15, 26, 27]. Here our findings demonstrated repetitive 20-Hz TI stimulation improved the height of CMJ and SJ. We speculate that this offline effect of TI stimulation is related to changes of motor cortical excitability, which might be dependent on spike-timing dependent plasticity [28, 29]. Transcranial magnetic stimulation (TMS) and functional magnetic resonance imaging (fMRI) studies have shown that externally beta oscillations (15–25 Hz) could increase cortical excitability and corticospinal excitability of M1 [30, 31], as well as alter the pattern of stimulated M1 connectivity [32, 33]. The positive correlation between the behavioral improvements and the increase in motor cortical excitability induced by 20-Hz TI stimulation has been reported in a previous TI study [15]. These findings may support our results that repetitive TI stimulation improved motor performance. Future neuroimaging studies investigating the functional changes of M1 induced by TI stimulation, which resulted in improved motor performance, are thus warranted.

However, it is interesting that there was no significant changes of maximum ground reaction force or impulse. Therefore, we speculated that the neurophysiological changes induced by TI stimulation did not lead to an increase in muscle strength or explosive power. Instead, it may affect motor control, athletic skills or muscle synergy, allowing participants to utilize their existing explosive power more efficiently, which may further be investigated by electromyographic (EMG). Conversely, Giustiniani et al. reported a negative effect of gamma-tACS on vertical jump performance, which suggested frequency might determine the modulation effect [34]. Additionally, considering the specific form of TI stimulation, we cannot exclude the influence of high-frequency stimulation on the motor cortex [6]. The neural response to kHz frequency stimulation is quite complex [9], indicating the importance of further elucidating the mechanisms underlying neuronal response to TI electric field on the motor cortex.

In our study, TI stimulation had no effect on the average CJ  height of last 15 s and fatigue index. Physical fatigue may result in movement-related beta decrease and post-movement beta rebound of the sensorimotor cortex [35]. However, the causal relationship between physical fatigue and brain oscillations remains unclear, which deserves further study to guide practical application. Another point worthy of notice is that cognitive-related regions are also involved in endurance exercise and mediating the anti-fatigue process [36, 37]. This viewpoint could be supported by a tDCS study, which demonstrated endurance performance improved after tDCS targeting dorsolateral prefrontal cortex, with the improvement in inhibitory control [38]. Therefore, our negative result may be attributed to the TI stimulation parameters in our study being unsuitable for the central control demands of endurance exercise. Furthermore, there were differential findings at different phases of CJ. At the beginning of CJ (first 15 s), there was a trend for improvement in average vertical jump height, with this trend disappearing in the final phase of CJ (last 15 s). This further supported our results that TI stimulation over M1 could improve transient explosive power performance but not fatigue resistance.

In the Y balance test, we found that 20-Hz TI stimulation over M1 did not improve the dynamic postural stability. Improvement in dynamic balance is not achieved simply by increasing the force output; instead, it involves the integration of different sensory systems, including visual, vestibular, and proprioceptive sensations [39]. Meanwhile, the central control of postural stability involves multiple cortical brain regions or networks, including the prefrontal-basal ganglia and sensorimotor networks [40, 41]. Previous studies on cortico-muscular coupling and cortical activity have confirmed the complexity of the intrinsic physiological mechanisms underlying human postural control [42, 43]. Taken together, the dynamic postural stability may not be substantially modulated by TI stimulation over M1.

This study has several limitations. First, this study lacked conventional NIBS techniques, such as tDCS or tACS, as a control group, which prevented the inference of differences between different technical approaches to clarify the regulatory advantages of TI stimulation. Second, we only explored the acute effects and did not track the long-term effects. Third, we did not employ neuroimaging tools (such as fMRI, electroencephalography, and functional near-infrared spectroscopy) to compare with the results of electrical field simulations. This comparison is essential for validating the actual stimulation target region and the neurophysiological effects of the stimulation protocol. Finally, we only selected uniform stimulation parameters. Considering individual variability, future studies should explore personalized stimulation sites and modulation parameters based on individual anatomy as well as functional and psychological statuses.

## Conclusion

This study demonstrated that repetitive TI stimulation over M1 leg area improved the vertical jump height of healthy adult males but did not alter anti-fatigue ability and dynamic postural stability. Although there is no neuroimaging evidence, our findings highlight the potential of TI stimulation in modulating motor abilities and provide evidence for further translational application of non-invasive deep brain stimulation. Future studies are warranted to elucidate the relationship between different TI parameters and behavioral performance in different populations (the elderly, athletes and patients), as well as to explore the underlying neural mechanisms.

## Data Availability

The datasets used and/or analysed during the current study are available from the corresponding author on reasonable request.

## Abbreviations

CMJ: Countermovement jump
CJ: Continuous jump
EEG: Electroencephalography
FI: Fatigue index
fMRI: Functional magnetic resonance imaging
fNIRS: Functional near-infrared spectroscopy
GRF: Ground reaction force
SJ: Squat jump
tACS: Transcranial alternating current stimulation
tDCS: Transcranial direct current stimulation
TI: Temporal interference
TMS: Transcranial magnetic stimulation
